# Precise Positioning of Myosin VI on Endocytic Vesicles In Vivo 

**DOI:** 10.1371/journal.pbio.0050210

**Published:** 2007-08-07

**Authors:** David Altman, Debanjan Goswami, Tama Hasson, James A Spudich, Satyajit Mayor

**Affiliations:** 1 Department of Biochemistry, Stanford University Medical Center, Stanford, California, United States of America; 2 National Centre for Biological Sciences, UAS-GKVK Campus, Bangalore, India; 3 Division of Biological Sciences, University of California San Diego, La Jolla, California, United States of America; Adolf-Butenandt-Institut, Germany

## Abstract

Myosin VI has been studied in both a monomeric and a dimeric form in vitro. Because the functional characteristics of the motor are dramatically different for these two forms, it is important to understand whether myosin VI heavy chains are brought together on endocytic vesicles. We have used fluorescence anisotropy measurements to detect fluorescence resonance energy transfer between identical fluorophores (homoFRET) resulting from myosin VI heavy chains being brought into close proximity. We observed that, when associated with clathrin-mediated endocytic vesicles, myosin VI heavy chains are precisely positioned to bring their tail domains in close proximity. Our data show that on endocytic vesicles, myosin VI heavy chains are brought together in an orientation that previous in vitro studies have shown causes dimerization of the motor. Our results are therefore consistent with vesicle-associated myosin VI existing as a processive dimer, capable of its known trafficking function.

## Author Summary

Myosin VI is a molecular motor implicated in diverse cell processes, including trafficking endocytic vesicles into the cell, transporting proteins to the leading edge of a migrating cell, and anchoring stereocilia to the hair cells of inner ear sensory epithelia. The motor has been studied in both a monomeric and dimeric form in vitro and is reported to exist as a monomer in the cytoplasm of cells. Because the functional characteristics of the motor are dramatically different for these two forms, an understanding of the activity of myosin VI requires an understanding of its functional form in vivo. To probe the role of myosin VI in vesicle trafficking, we labeled myosin VI truncations with a fluorescent protein and studied the positioning of these constructs on endocytic vesicles. We observed nonradiative transfer of energy between the fluorescent proteins, a process that can only occur if they are brought extremely close together. Our results indicate that, when myosin VI heavy chains bind to endocytic vesicles, they are precisely positioned very close together. Work from other laboratories indicates that myosin VI heavy chains brought together in this manner are capable of dimerization. Our results are therefore consistent with vesicle-associated myosin VI existing as a processive dimer, capable of myosin VI's known trafficking function.

## Abstract 3

Fluorescence anisotropy measurements are used to demonstrate that, on endocytic vesicles, myosin VI heavy chains are brought together in an orientation consistent with a processive dimer, capable of its known trafficking function.

## Introduction

Class VI myosins are found in a variety of organisms from Caenorhabditis elegans to human, and in a variety of cell types (reviewed in [1,2]). Unlike other characterized myosins, they move toward the pointed end of an actin filament [3], and so are capable of functions unique from other myosins. For example, during clathrin-mediated endocytosis, myosin VI is implicated in trafficking vesicles that have recently shed their clathrin coat, denoted uncoated vesicles (UCV). The motor transports UCV from the periphery of a cell to its interior, presumably along actin filaments in the cell periphery that are oriented with pointed ends directed toward the cell interior [4–6].

**Figure 1:**
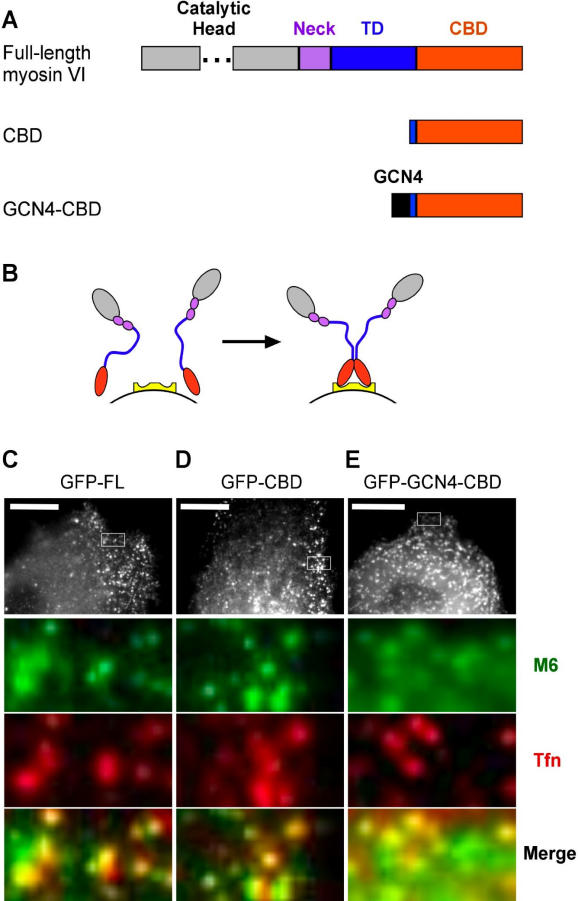
Myosin VI Constructs and Their Association with UCV (A) Cartoon depictions of the sequences for full-length myosin VI with TD in blue and CBD in orange, the myosin VI CBD containing 17 amino acids of the myosin VI TD (denoted simply as the CBD), and CBD with the addition of an N-terminal leucine zipper (GCN4-CBD). (B) Cartoon model depicting the precise positioning of myosin VI heavy chains on a UCV. The vesicle is represented by a black arc, and the yellow object represents an unknown protein to which the CBDs are binding. (C) The top image is the GFP fluorescence from an ARPE-19 cell expressing GFP-FL and endocytosing Alexa 647-labeled transferrin (Tfn). The white box contains the region of the cell shown in the images below the top image. The images below, from top to bottom, are the GFP fluorescence (M6), the Alexa 647 fluorescence (Tfn), and an overlay of the GFP and Alexa 647 fluorescence (Merge). GFP and Alexa 647 fluorescence have been made green and red, respectively. As a result, overlap between the two images results in yellow regions in the overlay image. Scale bar represents 17 μm. (D) These images are identical to those in (C), except that the ARPE-19 cell is expressing GFP-CBD while endocytosing Alexa 647-labeled Tfn. Scale bar represents 17 μm. (E) These images are identical to those in (C), except that the ARPE-19 cell is expressing GFP-GCN4-CBD while endocytosing Alexa 647-labeled Tfn. Scale bar represents 17 μm.

The motor's heavy chain contains an N-terminal catalytic head followed by a unique myosin VI insert and an IQ motif, each of which can bind a single calmodulin [7,8]. The calmodulin binding domains are followed by a tail domain (TD) that is predicted to be highly α-helical. The C-terminal domain is the motor's cargo-binding domain (CBD), a region implicated in association of the motor with its protein cargo [9–11] (Figure 1A).

Myosin VI heavy chains have been hypothesized to dimerize [7,12,13]. This model is supported by single-motor optical trap assays that utilized a motor construct containing a GCN4 leucine-zipper domain in the C-terminal region of the TD, ensuring dimerization of the motor even under the dilute (pM) conditions of single-molecule assays [14]. This dimer walks processively along actin, meaning it takes numerous steps along a filament before dissociating [15]. Its stepping is highly coordinated, with mechanical strain regulating the biochemical behavior of the molecule, resulting in head-to-head communication and proper in vivo function [16,17].

Surprisingly, however, Lister et al. [18] demonstrated that the myosin VI heavy chain, when purified from a baculovirus expression system or observed in extracts from rat kidney fibroblastic tissue culture, exists as a monomer. Though an ensemble of monomeric motors may be capable of myosin VI's predicted trafficking function, such an ensemble is not ideal for trafficking because the motor has a high duty ratio [16], and so monomers attached to actin would work against newly attached and stroking motors. On the other hand, as a coordinated processive dimer, the motor would be well suited to traffic cargo efficiently with relatively few motors, as demonstrated by in vitro studies of a myosin VI dimer [15,17]. We thus speculated that, in regions of the cell where myosin VI performs trafficking function, dimerization of the motor occurs in a regulated manner.

Park et al. [19] demonstrated that monomeric myosin VI motors lacking the CBD can dimerize in vitro if they are brought into close proximity, suggesting that myosin VI may be capable of in vivo dimerization in regions of high local motor concentration. However, the CBD appears to somewhat inhibit this dimerization, indicating that dimerization may require a proper positioning of the monomers.

Given the above considerations, we hypothesize that two myosin VI CBDs are precisely positioned close together when loaded onto a vesicle and that this positioning orients the motor appropriately for dimerization (Figure 1B). This would then allow the motor to perform its predicted trafficking function as a processive dimer [5].

Similar regulation of a motor protein between a monomer and dimer has been proposed for the C. elegans kinesin Unc104 [20,21]. However, mechanisms of dimerization for Unc104 and myosin VI are both inferred from in vitro data, and there is a lack of evidence indicating that this dimerization occurs in vivo. Here, we provide evidence for formation of a myosin VI dimer in vivo.

## Results/Discussion

### GFP-Tagged Full-Length Myosin VI Localizes to UCV

We conducted our studies in ARPE-19 cells, a human retinal pigment epithelial cell line that was one of the lines used by Dance et al. [6] in their in vivo studies of myosin VI. Dance et al. demonstrated in various cell lines that, during clathrin-mediated transferrin uptake, endogenous myosin VI colocalizes with transferrin-containing UCV [6]. Figure 1C shows a green fluorescent protein (GFP) image of an ARPE-19 cell expressing full-length myosin VI with an N-terminal GFP (GFP-FL). After transfection with the GFP-FL construct, cells exhibited two distributions of GFP fluorescence: a homogeneous GFP haze throughout the cytosol and small, bright GFP puncta that exist throughout the cell, though are often more dense in the cell periphery (Figure 1C). Dance et al. [6] observed similar colocalization for endogenous myosin VI.

To verify that the myosin VI puncta correspond to UCV, we observed endocytosis of transferrin conjugated with Alexa 647 dye (Alexa647-Tfn). Transferrin is known to be internalized via the clathrin-mediated endocytic pathway. GFP puncta showed a high degree of colocalization with internalized fluorescent transferrin immediately after internalization (Figure 1C).

### GFP-Tagged Myosin VI–CBD Constructs Localize to UCV Similarly to Full-Length Myosin VI

We next sought evidence for precise positioning of myosin VI motors on UCV. As discussed in the introduction, we speculated that myosin VI heavy chains, although likely to be monomeric in the cytoplasm [18], are brought into close proximity on its cargo, allowing the motor to function as a dimer.

Two primary myosin VI–truncated CBD constructs were used for these studies (Figure 1A). The first is a myosin VI containing the CBD as well as 17 residues from the TD N-terminal to the CBD (we refer to this simply as the CBD construct). The second construct is the CBD construct with a leucine zipper (GCN4) attached at its N-terminus (GCN4-CBD), which forces it to dimerize [14]. These constructs were made fluorescent by inserting a monomeric GFP isoform [22] at the N-termini of CBD (GFP-CBD) and of GCN4-CBD (GFP-GCN4-CBD).

After transfection of ARPE-19 cells with these constructs, cells exhibited the same two distributions of GFP fluorescence as cells transfected with GFP-FL: a homogeneous GFP haze throughout the cytosol and small, bright GFP puncta throughout the cell (Figure 1D and 1E). We also observed endocytosis of transferrin conjugated with Alexa 647 dye in these cells. GFP puncta showed a high degree of colocalization with internalized fluorescent transferrin immediately after internalization (Figure 1D and 1E). Thus, our constructs have maintained their ability to associate with UCV similarly to endogenous myosin VI [6].

In movies of cells expressing our GFP-tagged myosin VI–CBD constructs, UCV exhibited motion throughout the cell, with UCV toward the periphery of the cell typically exhibiting slower velocities relative to those further into the cell (see Videos S1–S3). For cells expressing GFP-FL, the slower motion likely corresponds to myosin VI–dependent movement of UCV through the thick actin-mesh at the cell periphery. This peripheral mesh is particularly thick in ARPE-19 cells, and UCV travel a net distance of approximately 2 μm through the actin in a process that takes on the order of 5 min [5]. For cells expressing myosin VI constructs lacking the catalytic head, the GFP construct competes with the endogenous motor for binding to the UCV, and acts as a dominant negative. In these cells, the slower motion corresponds to Brownian-like motion with a slow drift toward the interior of the cell [5]. The faster motion observed deeper in the cell may result from UCV moving on microtubules, consistent with predictions that a microtubule network is involved in intracellular trafficking of UCV from the early to late endosomes [23].

### HomoFRET of GFP-Tagged CBD Constructs Can Be Used to Probe for Precise Positioning of CBD on UCV

Fluorescence resonance energy transfer (FRET) is the nonradiative transfer of energy between fluorophores occurring when the emission spectrum of an excited fluorophore overlaps with the absorption spectra of a fluorophore in very close proximity (within 10–100 Å) [24]. FRET between identical fluorophores (homoFRET) serves as an ideal way for detecting homo-oligomeric protein configurations [25,26].

According to our proposed mechanism for myosin VI function when bound to its cargo (Figure 1B), the CBDs of UCV-associated myosin VI heavy chains are positioned to bring together the heavy chains. By analogy, this mechanism also predicts that, for two UCV-associated GFP-CBD constructs, the CBDs are positioned to bring into close proximity their associated GFPs. Thus, homoFRET of GFP-CBD on the UCV serves as a readout of our proposed mechanism.

GFP-GCN4-CBD serves as a positive control for detection of homoFRET. The leucine zipper forces the construct to form a constitutive dimer, resulting in close association of the GFPs adjacent to the GCN4 coiled coil and subsequent homoFRET. As a negative control, we used a construct that is identical to GFP-CBD, except the GFP is located at its C-terminus (CBD-GFP). For this construct, we expect that GFPs are not likely to be positioned to undergo homoFRET, even if the CBDs are brought close together.

**Figure 2:**
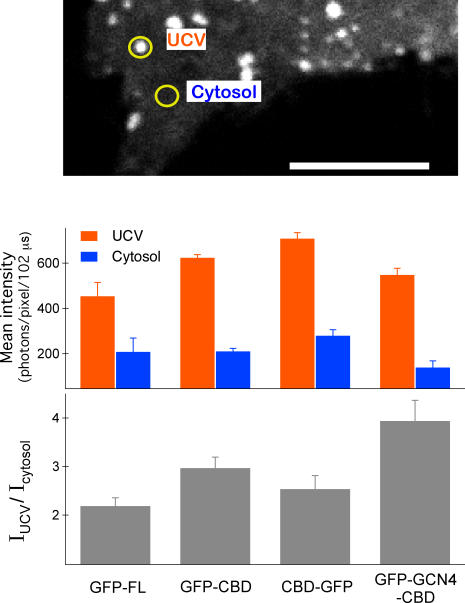
Steady-State Fluorescence Emission at UCV and in the Cytosol of Cells Expressing GFP-Tagged Myosin VI Constructs Top: confocal image of GFP fluorescence emission from an ARPE-19 cell expressing GFP-CBD. The yellow circles are representative regions of the cell that were selected for both steady-state and time-resolved fluorescence intensity and fluorescence anisotropy measurements. Scale bar represents 9 μm. Middle: mean steady-state GFP fluorescence emission at UCV (orange) and in the cytosol (blue) of ARPE-19 cells expressing GFP-tagged myosin VI constructs. For a particular cell expressing a GFP construct, the mean fluorescence emission at the UCV and in the cytosol were calculated from a confocal image of the steady-state fluorescence (see Materials and Methods). Approximately 20–60 UCV and 30 cytosol measurements were made for every cell. The fluorescence from *N* cells was then averaged to arrive at the values in the bar graph (GFP-FL, *N* = 7; GFP-CBD, *N* = 21; CBD-GFP, *N* = 12; GFP-GCN4, *N* = 12). Error bars indicate SEM. Bottom: ratio of mean GFP fluorescence emission intensity at the UCV and in the cytosol of ARPE-19 cells expressing GFP-tagged myosin VI constructs. Ratios are calculated from the values in the bar graph above, and error bars indicate the propagated errors.

To quantify the levels of GFP-FL, GFP-CBD, GFP-GCN4-CBD, and CBD-GFP in the cytosol and on UCV, we used multiphoton microscopy to gather confocal images of GFP fluorescence from ARPE-19 cells expressing these constructs. A sample image collected for GFP-CBD is shown in Figure 2 (top). Fluorescence emission was collected with multichannel plate photomultiplier tubes capable of photon counting. For an imaged cell, the mean photon count was calculated in numerous regions corresponding to the UCV and the cytosol (for example, see Figure 2, top), and these values were averaged to arrive at the cell's mean fluorescence intensity at each localization. The mean fluorescence intensities for multiple cells were then averaged to arrive at the overall mean fluorescence intensities at UCV and in the cytosol for each construct (see Materials and Methods). The total expression of all constructs was similar, as were their levels on UCV and in the cytosol (Figure 2, middle).

### Fluorescence Anisotropy Monitors HomoFRET and Rotational Diffusion of GFP Constructs

**Figure 3:**
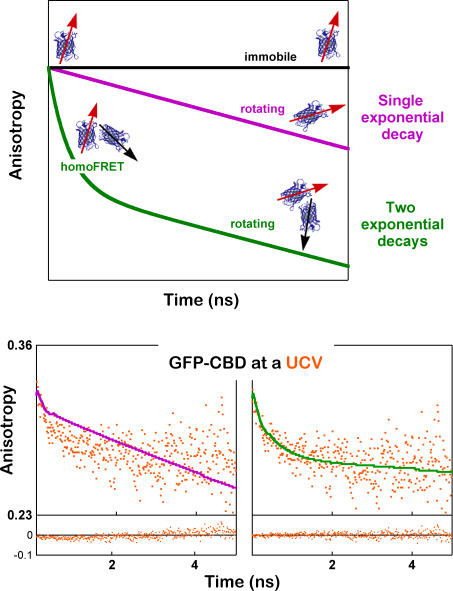
Expected Fluorescence Anisotropy Decays and Fits of Different Models to an Empirical Anisotropy Decay Profile Top: expected fluorescence anisotropy decay profiles for a fluorophore immobilized in an extremely viscous medium (black), tumbling in a less viscous medium (purple), or both tumbling and exhibiting homoFRET (green). Bottom: example of a fluorescence anisotropy decay profile measured at a UCV (orange points) for GFP-CBD. The decay was fit to a model describing decay through a single exponential process (purple) or through two exponential processes (green) (see Materials and Methods), and residuals are plotted below. The decay is not well fit by a single exponential decay (left), but is well fit by the model describing two exponential decays (right).

We excited the fluorophores of our GFP-tagged CBD constructs with pulsed, polarized excitation, and observed subsequent changes in fluorescence emission polarization, as quantified by the fluorescence anisotropy, over time. The emission polarization is initially aligned with the excitation polarization, resulting in a high initial anisotropy, and becomes randomized over the lifetime of the fluorophore through two processes: (1) rotational diffusion of the GFPs and (2) energy transfer to GFPs in close proximity (on the order of the Förster's radius) [27]. Rotational diffusion and homoFRET each result in exponential decays in anisotropy which, for large proteins, occur on very different time scales [26,28]: homoFRET results in a rapid anisotropy decay, and rotational diffusion results in a slower decay (Figure 3, top). From the former, we can detect processes that bring GFPs into close proximity, and from the latter, we can infer the size of the rotating object.

We measured fluorescence anisotropy following polarized multiphoton excitation with a pulsed laser (∼12-ns repetition rate) using time-correlated single-photon counting (TCSPC) [26,29] (see Materials and Methods). Pico-second time-resolved anisotropy decays were measured for our three GFP-tagged CBD constructs in the cell periphery, both in the cytosol and at the UCV. An example of the regions selected for these measurements is shown in Figure 2 (top). We selected UCV in the cell periphery to be sure the construct is associated with UCV in the peripheral actin network. To further ensure that we selected for these vesicles, we collected an image encompassing the area of the measured UCV both before and after the measurement, an interval lasting approximately 1 min. UCV that remained in the observation volume before and after the measurement corresponded to slowly moving UCV that were associated with the peripheral actin network and so were selected for analysis.

**Figure 4:**
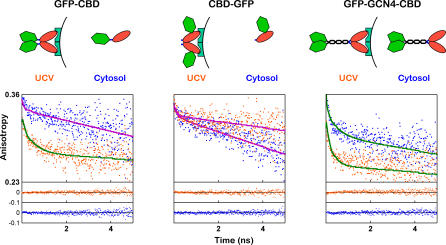
Time-Resolved Fluorescence Anisotropy for GFP-Tagged Myosin VI Constructs at UCV and in the Cytosol Cartoon models depict the functional forms of GFP-CBD (top left), CBD-GFP (top middle), and GFP-GCN4-CBD (top right) on a UCV and in the cytosol. Below the cartoons are examples of fluorescence anisotropy decay profiles measured at a UCV (orange points) and in the cytosol (blue points) for each construct. The decays are fit either to a model describing decay through a single exponential process (purple) or through two exponential processes (green) (see Materials and Methods), and residuals are plotted below the curves.

We fit two decay models to each empirical anisotropy decay: (1) a single exponential decay and (2) the sum of two exponential decays (see Materials and Methods and Figure 3). These fits revealed two classes of decay profiles. For the first class, the profiles were well fit by a single exponent; the addition of another exponent had little effect on the fit. These profiles describe decay in anisotropy through only a single process, presumably rotational diffusion. For a second class, the decay was not fit well by a single exponent, but the addition of a second exponent resulted in a good fit (for example, see Figure 3, bottom). These profiles describe decay in anisotropy through two exponential processes, both homoFRET and rotational diffusion. In this manuscript, we describe in detail the best fits for all empirical decays, the first class of decays to a single exponent and the second class to the sum of two exponents (Figure 4 and Table 1).

**Table 1:**
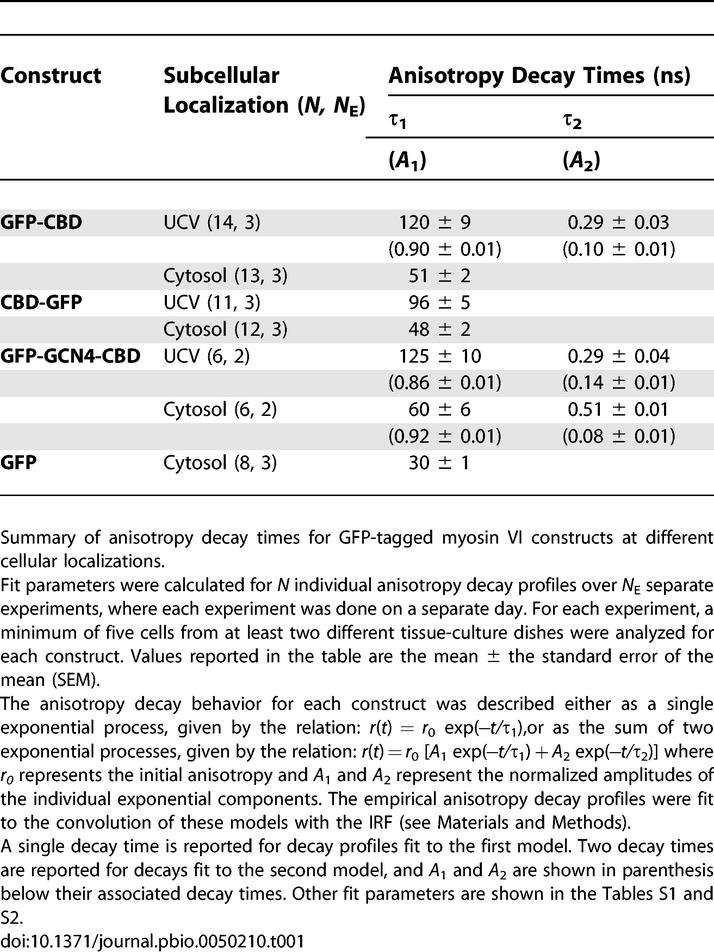
Anisotropy Decay Times of GFP-Tagged CBD Constructs

As a control to test the instrumentation, we transfected cells with monomeric GFP. The GFP homogeneously filled the cytoplasm, and anisotropy decay profiles collected from cytosolic GFP were well fit by a single exponent (Figure S1). The time scale of this decay (∼25 ns) is consistent with previous measurements of GFP tumbling in the cytosol [30].

### CBD-GFP Does Not Exhibit HomoFRET in the Cytosol or at UCV

For CBD-GFP, anisotropy decays measured both in the cytosol and at UCV were well fit by a single exponent (Figure 4, middle), consistent with our expectation that the construct does not undergo homoFRET and that anisotropy decreases only through fluorophore rotation. The time scale of this decay in the cytosol is consistent with tumbling, and the decay is considerably slower at the UCV, consistent with a slowed rotation due to association of the CBD-GFP with a UCV (Table 1).

### GFP-GCN4-CBD Exhibits HomoFRET in the Cytosol and at UCV

Anisotropy decays collected for GFP-GCN4-CBD both in the cytosol and at UCV could not be fit by a single exponential but were well fit by the sum of two exponents (Figure 4, right), consistent with our expectation that anisotropy decreases both through rotational diffusion and through homoFRET of the dimeric construct. The time scales for the fast and slow decays are consistent with homoFRET and tumbling, respectively. As with CBD-GFP, the decay corresponding to rotation is slower at the UCV relative to the cytosol due to association with the UCV (Table 1).

### GFP-CBD Exhibits HomoFRET at UCV but Not in the Cytosol

Anisotropy decays collected from GFP-CBD in the cytosol were well fit by a single exponent. Anisotropy decays collected at UCV, however, were only well fit by the sum of two exponents (Figure 3, bottom, and Figure 4, left). The rapid anisotropy decay at UCV is consistent with a homoFRET process. Both the slower decay at the UCV and the decay in the cytosol are consistent with rotational diffusion. As with the other GFP-constructs, the decay describing rotation is slower at the UCV compared to the cytosol (Table 1).

From these data, we infer that GFP-CBDs are positioned on UCV to bring their N-termini together (Figure 1B). The lack of homoFRET in the cytosol confirms that this precise positioning requires the construct to be loaded onto the vesicle. This result is consistent with our prediction that a precise positioning of CBDs on a vesicle orients heavy chains in close proximity.

### GFP-CBD Rotates as a Monomer in the Cytosol

For anisotropy decays measured in the cytosol, the time scale describing rotational diffusion (Table 1) provides information about the size of the GFP construct. Because these decay times are longer than the fluorescence lifetime of GFP (∼3 ns), unambiguous molecular weights cannot be determined. However, we can infer relative sizes of our constructs from these decays.

Rotation of GFP-CBD is slower than for GFP alone (Figure S1), consistent with slowed rotational diffusion of the fluorophore when attached to the myosin VI construct. The 2-fold difference in the decay time suggests that the molecular weight of GFP-CBD is twice that of GFP (molecular weight, 27 kDa), consistent with a monomeric form of GFP-CBD (molecular weight, 58 kDa).

The rotational decay time of GFP-CBD is also similar to that of CBD-GFP, and both constructs rotate faster than GFP-GCN4-CBD (Figure S1). Thus, both GFP-CBD and CBD-GFP appear to be smaller than a similarly sized GFP construct known to dimerize, supporting our prediction that both are monomeric in the cytosol.

### Steady-State Anisotropy Measurements Provide Further Evidence for HomoFRET of GFP-CBD at UCV

**Figure 5:**
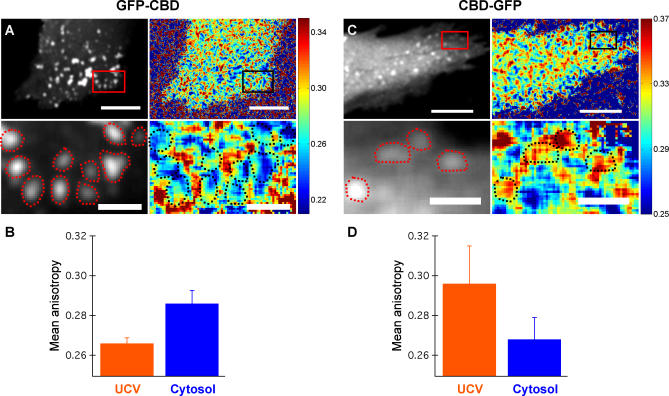
Steady-State Fluorescence Emission and Anisotropy of Cells Expressing GFP-CBD and CBD-GFP (A) Steady-state GFP fluorescence emission intensity images (left) and anisotropy images (right) for a cell expressing GFP-CBD. Rectangles in the upper images indicate the region displayed in the lower images. To reduce noise, two–nearest-neighbor averaging was applied to the parallel- and perpendicular-polarization emission intensity images before calculating the total emission intensity and anisotropy images (see Materials and Methods). The units of the total intensity image are in photons/pixel, where photon count is integrated over the pixel residence time of the measurement (102 μs for all measurements). A color map was applied to the anisotropy image and is shown to the right of the images. To improve contrast of the fluorescence emission image, intensities greater than 2,000 photons/pixel have been set to 2,000 for the upper image and intensities greater than 1,600 photons/pixel have been set to 1,600 for the lower image. To improve contrast of the anisotropy image, anisotropies higher than 0.35 and lower than 0.21 have been set to 0.35 and 0.21, respectively. Regions of high intensity in the emission-intensity image are circled with red dashed lines, and the identical regions in the anisotropy image are circled with black dashed lines. Scale bar represents 10 μm for the upper images, and 2 μm for the lower images. (B) For the cell in (A), steady-state anisotropy was calculated at regions corresponding to GFP-CBD both at the UCV and in the cytosol, and the mean anisotropy at both localizations is plotted. Mean anisotropy at the UCV and in the cytosol are 0.266 ± 0.003 (*n* = 58, ± SEM) and 0.286 ± 0.007 (*n* = 30, ± SEM), respectively. (C) Steady-state GFP fluorescence emission intensity images (left) and anisotropy images (right) for a cell expressing CBD-GFP. The same presentation is used as in (A). To improve contrast of the fluorescence emission image, intensities greater than 850 photons/pixel have been set to 850 for the upper image and intensities greater than 450 photons/pixel have been set to 450 for the lower image. To improve contrast of the anisotropy image, anisotropies higher than 0.37 and lower than 0.25 have been set to 0.37 and 0.25, respectively. Scale bar represents 10 μm for the upper images, and 2 μm for the lower images. (D) For the cell in (C), steady-state anisotropy was calculated at regions corresponding to CBD-GFP both at the UCV and in the cytosol, and the mean anisotropy at both localizations is plotted. Mean anisotropy at the UCV and in the cytosol are 0.296 ± 0.019 (*n* = 15, ± SEM) and 0.269 ± 0.011 (*n* = 30, ± SEM), respectively.

To further demonstrate that homoFRET occurs when GFP-CBD is associated with UCV, we determined both the steady-state fluorescence emission and steady-state fluorescence anisotropy throughout cells expressing GFP-CBD (Figure 5A). Steady-state anisotropy represents the integral over time of an anisotropy decay profile, and so it is reduced by both rotational diffusion and homoFRET (Note the areas under the curves in Figure 3, top), though these processes cannot be distinguished by steady-state analysis [31]. In all cells analyzed, steady-state anisotropy for GFP-CBD was clearly lower at UCV relative to the surrounding cytosol (Figure 5A). Considering only the effects of fluorophore tumbling, we would have expected steady-state anisotropy to be lower in the cytosol, where rotational diffusion is more rapid. The observed pattern of steady-state anisotropy is thus consistent with a further reduction in steady-state anisotropy at the UCV due to homoFRET.

To quantify this, we manually selected from the periphery of each cell numerous UCV as well as 30 regions in the cytosol, similar in size to the UCV, and calculated the steady-state anisotropy at these regions (see Materials and Methods). We observed that the mean steady-state anisotropy of these regions at either localization is not consistent from cell to cell, due to a variety of factors such as cell thickness. However, for all ten cells analyzed, we observed that the mean anisotropy at the UCV was consistently lower than the mean anisotropy in the cytosol. An example of analysis of a single cell is shown in Figure 5B.

As a control, we also determined the steady-state fluorescence anisotropy and emission throughout cells expressing CBD-GFP. Because CBD-GFP does not exhibit homoFRET at UCV, its steady-state anisotropy should be dictated solely by its rotational diffusion. Thus, we expect a higher anisotropy at UCV relative to the surrounding cytosol, in contrast to the pattern observed for cells expressing GFP-CBD. We observed this expected pattern of steady-state anisotropy for all cells analyzed (Figure 5C), and again we quantified this by calculating steady-state anisotropy at numerous UCV regions and at 30 regions corresponding to the cytosol. For all nine cells analyzed, we observed that the mean steady-state anisotropy at the UCV was higher than the steady-state anisotropy in the cytosol. An example of analysis of a single cell is shown in Figure 5D.

### HomoFRET of GFP-CBD Does Not Result from Crowding

Our time-resolved and steady-state anisotropy experiments demonstrated that GFP-CBD undergoes homoFRET when localized to UCV. Though we hypothesize that this is the result of precise positioning of the construct on a UCV, we must also consider the possibility that GFPs are brought into close proximity simply due to the crowding of a high density of GFP-CBD on the UCV surface.

From our analysis of steady-state GFP-fluorescence emission images of cells expressing GFP-CBD and CBD-GFP, we observed that both constructs are expressed to similar levels in our cell line (Figure 2, middle). Furthermore, both constructs exhibit similar ratios of GFP intensities on UCV and in the cytosol, indicating that they are loaded onto vesicles at similar densities and so are similarly crowded (Figure 2, bottom). Thus, if homoFRET of GFP-CBD were the result of crowding, we would also expect CBD-GFP to exhibit homoFRET when loaded onto UCV. Since this is not the case (Figure 4, middle), crowding cannot be the cause of homoFRET. Instead, the CBD must be positioned on a UCV so that its N-termini are brought together, resulting in homoFRET from an N-terminal (and not a C-terminal) GFP.

We confirmed this conclusion by examining UCV associated with varying densities of GFP-CBD. If homoFRET were the result of crowding, then reducing the construct concentration on the UCV would reduce close packing of fluorophores and subsequently reduce the occurrence of homoFRET. On the other hand, if homoFRET results from precise positioning of GFP-CBD on UCV, then, even at low densities, the construct will undergo homoFRET.

To differentiate between these mechanisms, we calculated the steady-state fluorescence emission intensity at the UCV regions selected from the previous steady-state anisotropy analysis (see Figure 5 and Materials and Methods). Using these measurements, we probed for effects of GFP-CBD density on homoFRET by looking for effects of varying steady-state fluorescence emission intensity on steady-state fluorescence anisotropy (Figure S2). To determine the degree to which these measured values of anisotropy and intensity are related, we calculated the Pearson product-moment correlation coefficient *(r)*.

For ten cells expressing GFP-CBD, the UCV regions of nine cells showed no significant correlation between steady-state fluorescence emission intensity and anisotropy (*p* > 0.05 for nine cells, *p* = 0.02 for one cell). The lack of correlation indicates that the extent of homoFRET does not depend on the density of UCV-associated GFP-CBD. This supports our conclusion that homoFRET results from precise positioning of GFP-CBD on the vesicle, and not crowding of the fluorophores.

As expected, when we performed a similar analysis for GFP-CBD regions in the cytosol, where the construct does not undergo homoFRET, we observed no correlation between fluorescence emission intensity and anisotropy. The same is true for the UCV and cytosolic regions of cells expressing CBD-GFP, which does not undergo homoFRET at either localization, and for GFP-GCN4-CBD, which undergoes homoFRET at both localizations due to precise positioning of its fluorophores (unpublished data).

In summary, understanding the in vivo functional form of a molecular motor is essential to understanding its function. Our data suggest that, although myosin VI exists as a monomer in the cytosol, heavy chains are brought into close proximity on UCV, allowing the motor to function as a dimer. Consistent with our model, Spudich et al. [13] reported that a myosin VI tail construct, when bound to artificial lipid vesicles in vitro, can be linked as dimers upon addition of a zero-length cross-linker. Through this mechanism, myosin VI is able to processively traffic its vesicular cargo through the actin meshwork in the cell periphery [32].

## Materials and Methods

### DNA constructs.

GFP constructs were derived from the GFP-HM6Tail+LI construct from Dance et al. [6], which consists of a myosin VI–CBD construct in the pEGFP-C3 expression vector (Clontech, http://www.clontech.com). GFP-CBD was made from GFP-HM6Tail+LI by changing residue 206 of the GFP from alanine to lysine *(A206K),* which reduces the proclivity of GFP to dimerize [22]. This was achieved through site-directed mutagenesis using the primer 5′-CCTGAGCACCCAGTCCAAGCTGAGCAAAGACCCCA-3′ and the QuikChange Site-Directed Mutagenesis Kit (Stratagene, http://www.stratagene.com).

To make GFP-GCN4-CBD, the leucine zipper from the myosin VI/GFP plasmid described in [33] was amplified using the primers 5′-CCCGAATTCTGGAAGACATGAAACAGCTCGAGGACAAAGTAGAGGAGCTGCTGTCCAAG-3′ and 5′-GCCCGCGGCTCCCCGACCAGCTTCTTAAGTCTCGCAACCTCATTTTCTAGATGG-3′.

The resulting PCR product was cut with EcoRI and SacII, and inserted into the MCS of the GFP-CBD plasmid.

To make CBD-GFP, CBD was amplified from the GFP-CBD plasmid using the primers 5′-CGCCGCGGATGAGGATTGCCCAGAGTGAAGCCGAGCTCATCAGTGATGAGGCCC-3′ and 5′-TTGGATCCGCCTTTAACAGACTCTGCAGCATGGCTGTTGCATAGGTGGGCCGAGCCTG-3′.

The resulting PCR product was cut with SacII and BamH1, and inserted into the multiple cloning site (MCS) of the pEGFP-N1 expression vector (Clontech) containing the *A206K* GFP mutation. The *A206K* mutation was made in pEGFP-N1 using the site-directed mutagenesis described above.

### ARPE-19 cell culture and protein transfection.

ARPE-19 cells were purchased from American Type Culture Collection (ATCC, http://www.atcc.org). Cells were grown at incubating conditions (37 °C and 5% CO_2_) in medium + serum (DMEM/F-12 [GIBCO-Invitrogen, http://www.invitrogen.com], 1% fungizone [GIBCO], 1% L-glutamate [GIBCO], 10% FBS [GIBCO], 1.5 M HEPES, 100 U/ml penicillin, and 100 mg/ml streptomycin). Before transfection, cells were grown in imaging dishes that were polylysine-coated, with a translucent bottom appropriate for fluorescence imaging. To transfect cells, a transfection mixture, consisting of 75 μl of serum-free media (SFM; medium+serum lacking FBS), 6 μl of TransIT Transfection Reagent (Mirus Bio Corporation, http://www.mirusbio.com), and 1 μg of plasmid DNA, was added to the cell culture, which is in 0.75 ml of medium + serum. Cells were imaged 10–20 h after transfection.

### Transferrin uptake in ARPE-19 cells.

Transferrin was labeled with Alexa 647 (Alex647-Tfn) using the Alexa Fluor 647 Protein Labeling Kit (Molecular Probes, http://probes.invitrogen.com). To observe uptake of Alexa647-Tfn, cells grown in imaging dishes were starved in SFM for 2 h at incubating conditions. The media was removed, and 150 μl of 10 μg/ml Alexa647-Tfn in SFM was applied to the cells. The cells were left at incubating conditions for 30 min.

Cells were fixed by washing in M1 buffer (150 mM NaCl, 5 mM KCl, 1 mM MgCl_2_, 1 mM CaCl_2_, 20 mM HEPES [pH 7.4]) and then adding 150 μl of 4% paraformaldehyde in M1 buffer. Cells were incubated for 25 min at room temperature, washed with M1 buffer, and imaged.

For colocalization experiments, the transferrin-internalization protocol was begun 10.5 h after transfection of cells with the GFP construct.

### Wide-field imaging.

Briefly, experiments were done on a Nikon TMD fluorescence microscope with a cooled back-illuminated, 16-bit charge-coupled device (CCD) camera (Nikon, http://www.nikonusa.com). Different filter sets were used to image Alex 647 and GFP. Images were collected using Metamorph software (Molecular Devices, http://www.moleculardevices.com). Fluorescence imaging was carried out exactly as described [34].

### Steady-state and time-resolved anisotropy measurements using multiphoton excitation.

Live-cell measurements of fluorescence anisotropy were made using TCSPC and pulsed multiphoton excitation. Details of the method and analysis will be described elsewhere (D. Goswami, K. Gowrishankar, M. Rao, and S. Mayor, unpublished data). Briefly, steady state and time-resolved anisotropy measurements of fluorophores excited by multiphoton excitation were made on a Zeiss LSM 510 Meta microscope (Carl Zeiss, http://www.zeiss.com) with 63× 1.4 numerical aperture (NA) objective coupled to the femtosecond-pulsed Tsunami Titanium:Sapphire tunable pulsed laser (Newport, http://www.newport.com). Parallel and perpendicular emissions were collected simultaneously into two Hamamatsu R3809U multi-channel plate photomultiplier tubes (PMTs; Hamamatsu Photonics, http://www.hamamatsu.com) using a polarizing beam splitter (Melles Griot, http://www.mellesgriot.com) at the non-descanned emission side. TCSPC was accomplished using a Becker & Hickl 830 card (Becker and Hickl, http://www.becker-hickl.de), operating in a stop–start configuration [35]. For multiphoton excitation of GFP or fluorescein in cells, we used 920-nm excitation wavelength. At this wavelength, the two-photon absorption cross section for GFP is higher, enabling lower laser excitation power, and autofluorescence signals are minimized. The repetition rate of the pulsed laser is 80.09 MHz (12 ns).

Steady-state imaging was accomplished using a pixel residence time of 102 μs/pixel, setting the detection time resolution in the Becker and Hickl card to one. Thus, a full image (512 × 512 pixel) was collected over 62 s. For time-resolved anisotropy measurements, the time resolution was 12.2 ps. The beam was “parked” at a single point using routines available in the Zeiss software. The parked beam was placed at the center of the field to maintain uniformity of G-Factor, and photons were collected for 30–50 s. Photons were collected at a maximum rate of 0.1 MHz to ensure that TCSPC conditions were strictly met. Because of the low laser power, less than 10% bleaching was observed during a measurement. The instrument response function (IRF) was measured using 10–16-nm gold particles dried on a coverslip as a second harmonic generator; full width at half maximum (FWHM) of IRF is approximately 60 ps.

In our experimental setup, the steady-state anisotropy measured while the laser beam is parked at a single point (Table S2) was always higher than the steady-state anisotropy measured using the scanning mode (Figure 5B and 5D). This is attributed to a small but detectable depolarization of the excitation laser beam in the scanning mode when using high NA objectives, which is also seen for measurements of a monomeric GFP solution. This effect is negligible for objectives with NA less than 0.8 (unpublished data). A high NA objective was required to discern GFP associated with UCV as puncta distinct from the GFP cytosolic haze.

### Fits of time-resolved anisotropy measurements to decay models.

Fluorescence lifetime and anisotropy decay analyses were done essentially as described [26,36,37], with minor modifications in the analysis procedure. Briefly, the experimentally measured fluorescence decay is a convolution of the IRF with the intensity decay function. The intensity decay data were fit to the appropriate equations by an iterative reconvolution procedure using a Levenberg-Marquardt minimization algorithm.

When fitting the models to the decay profiles, *r*_o_ was constrained to a small window to improve the ability of the fitting algorithm to find the optimal fit. A constrained range of values for *r*_o_ (0.43 ± 0.3) was used for all fits described in the manuscript. This range of values was obtained from unconstrained fitting of cytoplasmically expressed GFP fluorescence emission anisotropy decays (*n* = 8). These fits provided reliable values for the initial anisotropy because the fluorophore does not undergo homoFRET and because the time scale of rotation is much slower than the time scale of the measurement.

When fitting a model describing two exponential decays to the decay profiles, the two decay times were somewhat constrained to a wide range of values. These constraints involved large windows centered on the expected decay times for the physical processes involved (homoFRET and rotational diffusion). Again, these constraints improved the ability of the fitting algorithm to find the optimal fit.

It is important to note that the empirical anisotropy decay profile is the convolution of the real-time behavior of the fluorophore with the IRF. This distortion results in an apparent fast decay at the start of all measurements that is an artifact and does not represent anything physical. This artifact is apparent because our sampling rate of 12.2 ps is smaller than the width of the instrument's IRF (∼60 ps). This effect is also apparent in the empirical decay for a monomeric GFP in the cytoplasm (Figure S1).

The G-Factor was estimated using a fluorescein solution and setting the anisotropy at late times to 0.005. Fluorescence and anisotropy decays were considered well fit if three criteria were met: reduced χ^2^ was less than 1.4, residuals were evenly distributed across the full extent of the data, and visual inspection ensured that the fit accurately described the decay profile.

### Averaging steady-state images.

For analysis of steady-state images, *N* nearest-neighbor averaging of a 512 × 512 array of pixel values refers to the following calculation: the pixel value at location (row = *i,* column = *j*) was set to the mean value of pixels spanning rows *i* − *N* to *i* + *N* and columns *j* – *N* to *j* + *N*. This calculation was done using software developed in Matlab (The MathWorks, http://www.mathworks.com).

### Analysis of steady-state anisotropy data.

Steady-state anisotropy was calculated from steady-state parallel- and perpendicular-polarization images. To account for differences in the optical paths traversed by the perpendicular and parallel emissions, a G-Factor correction was applied to the data as follows. We collected steady-state emission images from a fluorescein sample. Because fluorescein tumbles rapidly relative to the time scale of our measurements, fluorescein provides a pixel-by-pixel readout of the detector output from a source emitting identically in both polarizations. We created a G-Factor image from the parallel and perpendicular fluorescein emission images, and perpendicular images from subsequent experiments were multiplied by this G-Factor image to apply the appropriate correction.

To create this G-Factor image, dividing the parallel and perpendicular fluorescein images pixel by pixel is insufficient, and results in a correction that is artificially too large. This is because pixel values in the parallel and perpendicular images exhibit Poisson photon noise. To reduce artifacts arising from dividing signals containing noise, the images must first be averaged so as to increase the signal-to-noise ratio. Using simulated data, we determined the extent of averaging required to sufficiently reduce the G-Factor artifact while retaining information about G-Factor variation across the image (unpublished data). To create our G-Factor image, we first applied three-nearest-neighbor averaging to the parallel and perpendicular fluorescein images and then divided the averaged images pixel by pixel. A new G-Factor image was created for each day of experiments.

After applying the G-Factor correction to the data, anisotropy was calculated at UCV and in cytosolic regions using software developed in ImageJ [38]. Regions were manually selected in the parallel image and were transferred to the perpendicular image. The mean perpendicular- and parallel-polarization emission intensities were calculated for each region, and from these, the steady-state fluorescence anisotropy and fluorescence emission intensity were calculated using the relations:

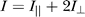
and

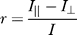
where *I*_‖_ and *I*_⊥_ are the calculated intensities of fluorescence emission with polarization parallel and perpendicular to the excitation polarization, respectively, *I* is the total fluorescence emission intensity, and *r* is the fluorescence anisotropy.

## Supporting Information

Figure S1 Fluorescence Anisotropy Decays for GFP and CBD Constructs in the Cytosol Anisotropy decay profiles for GFP, GFP-CBD, CBD-GFP, and GFP-GCN4-CBD measured in the cell cytosol are shown below the cartoon of each construct. The decays have been fit either to a model describing decay through a single exponential process (purple line) or through two exponential processes (green line). Residuals for the fits are shown below. Note that all three decays exhibit a very rapid decay in anisotropy at very short time scales (<100 ps) that is more rapid than decay in anisotropy due to homoFRET (for example, see the ∼500-ps homoFRET decay in the GFP-GCN4-CBD anisotropy decay profile). This less than 100-ps decay is an artifact resulting from the IRF (see Materials and Methods). Also note that rotation of the GFP is the most rapid, whereas rotation of GFP-GCN4-CBD (as described by the anisotropy decay for times >2 ns) is the slowest.
(881 KB TIF)

Figure S2 Steady-State Anisotropy and Fluorescence Emission Intensity at UCV for a Cell Expressing GFP-CBD Plots of steady-state anisotropy as a function of steady-state fluorescence emission intensity for UCV regions selected from the periphery of a cell expressing GFP-CBD. The units of total intensity are in photons/pixel, where photon count is integrated over the pixel residence time of the measurement (102 μs for all measurements). The mean anisotropy is 0.270 ± 0.025 (*n* = 63, ± standard deviation [SD]) and is indicated by the orange dashed line. There is no significant correlation between intensity and anisotropy, as judged by Pearson's *r*-value (*r*(63) = 0.114, *p* > 0.05).
(217 KB TIF)

Table S1Fluorescence Decay Times of GFP-Tagged CBD-ConstructsSummary of fluorescence anisotropy decay fit parameters for GFP-tagged myosin VI constructs at different cellular localizations. Fit parameters were calculated for *N* individual anisotropy decay profiles over *N*_E_ separate experiments, where each experiment was done on a separate day. For each experiment, a minimum of five cells from at least two different tissue-culture dishes were analyzed for each construct. Values reported in the table are the mean ± the standard error of the mean (SEM).Anisotropy decay profiles were either fit to a model describing decay through a single exponential process, given by the relation:

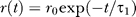
or a model describing decay through two exponential processes, given by the relation:

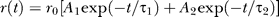
where *r*_0_ represents the initial anisotropy and *A*_1_ and *A*_2_ represent the normalized amplitudes of the individual exponential components.
Additional fit parameters include the steady-state anisotropy (*r*_ss_) and the parameters describing decay of GFP fluorescence intensity. GFP fluorescence intensity decay was well described by the relation:

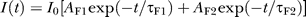
where *I*_0_ is the initial fluorescence emission intensity and *A*_F1_ and *A*_F2_ represent the normalized amplitudes of the individual exponential components. The two decay times are presented in the table with *A*_F1_ and *A*_F2_ in parenthesis below.
The fluorescence intensity decay for GFP is known to be well described by the sum of two exponentials, though previous work reported decay times that differ from our observed values. For example, Volkmer et al. [29] measured decay times of 0.17 ns and 2.8 ns. These differences likely result from the differences between the GFP variant used by Volkmer et al. and the variant used for this work.It should be noted that the fit values of *r*_0_ in the table (∼0.44) are larger than the initial anisotropies observed in the empirical decay profiles (Figure 4). This apparently reduced initial anisotropy observed in the decay trace is a consequence of the experimental method. It occurs for the following reason: the sample is excited with pulsed polarized excitation every 12 ns, and the time-resolved anisotropy is measured during the 12-ns interval between pulses. However, after a 12-ns interval, not all excited fluorophores have returned to a ground state. Emission from these fluorophores thus “rolls over” to the net emission from the next excitation pulse. Fluorophores that have rolled over have a fluorescence emission that is relatively depolarized due to their rotational diffusion. Thus, this population of fluorophores lowers the initial anisotropy observed in our decays. When fitting anisotropy decay models to the data, we have taken into account this roll-over effect.Reduced χ^2^ values for the fits, which were used to validate the goodness of individual fits, are also reported.(36 KB DOC)

Table S2 Additional Anisotropy Decay Parameters of GFP-Tagged CBD-Constructs See Table S1 legend for more detailed information.
(37 KB DOC)

Video S1 Movie of an ARPE-19 Cell Expressing GFP-CBD. The video consists of 61 images, captured consecutively. Each image is a 3-s exposure. Images are 15 μm by 20 μm.
(7.4 MB AVI)

Video S2 Movie of an ARPE-19 Cell Expressing GFP-GCN4-CBD. The video consists of 61 images, captured consecutively. Each image is a 3-s exposure. Images are 15 μm by 20 μm.
(12.3 MB AVI)

Video S3 Movie of an ARPE-19 Cell Expressing CBD-GFP. The video consists of 61 images, captured consecutively. Each image is a 3-s exposure. Images are 10 μm by 20 μm.
(7.8 MB AVI)

## Abbreviations

CBD: cargo-binding domain
GFP: green fluorescent protein
homoFRET: fluorescence resonance energy transfer between identical fluorophores
IRF: instrument response function
NA: numerical aperture
TCSPC: time correlated single-photon counting
TD: tail domain
UCV: uncoated vesicles
