# Supplementary material for: Precise Positioning of Myosin VI on Endocytic Vesicles In Vivo
Source: PLoS Biol. 2007 Aug 7;5(8):e210. doi: 10.1371/journal.pbio.0050210 (PMC1939883; doi:10.1371/journal.pbio.0050210)
Supplement: Supplementary file 1 [file pbio.0050210.st001.doc]

**Supplemental Table S1: Fluorescence decay times of GFP-tagged CBD-constructs**

|  | Sub-cellular  localization (N, N_E_) | Fluorescence decay times (ns) | |
| --- | --- | --- | --- |
|  |  | *τ_F1_*  (*A_F1_*) | *τ_F2_*  (*A_F2_*) |
| GFP-CBD | UCV (14, 3) | 2.13 ± 0.02  (0.62 ± 0.02) | 0.49 ± 0.01  (0.38 ± 0.02) |
|  | Cytosol (13, 3) | 2.29 ± 0.06  (0.57 ± 0.03) | 0.51 ± 0.03  (0.43 ± 0.03) |
| CBD-GFP | UCV (11, 3) | 2.15 ± 0.02  (0.66 ± 0.02) | 0.46 ± 0.01  (0.34 ± 0.02) |
|  | Cytosol (12, 3) | 2.31 ± 0.02  (0.69 ± 0.02) | 0.56 ± 0.03  (0.31 ± 0.02) |
| GFP-GCN4-CBD | UCV (6, 2) | 2.03 ± 0.02  (0.59 ± 0.03) | 0.43 ± 0.02  (0.41 ± 0.03) |
|  | Cytosol (6, 2) | 2.35 ± 0.05  (0.59 ± 0.03) | 0.56 ± 0.02  (0.41 ± 0.03) |
| GFP | Cytosol (8, 3) | 2.72 ± 0.04  (0.70 ± 0.03) | 0.64 ± 0.09  (0.30 ± 0.03) |
