# Supplementary material for: Precise Positioning of Myosin VI on Endocytic Vesicles In Vivo
Source: PLoS Biol. 2007 Aug 7;5(8):e210. doi: 10.1371/journal.pbio.0050210 (PMC1939883; doi:10.1371/journal.pbio.0050210)
Supplement: Supplementary file 2 [file pbio.0050210.st002.doc]

**Supplemental Table S2: Additional anisotropy decay parameters of GFP-tagged CBD-constructs**

|  | Sub-cellular  localization (N, N_E_) | *r_o_* | *r_ss_* | *χ^2^* |
| --- | --- | --- | --- | --- |
| GFP-CBD | UCV (14, 3) | 0.43 ± 0.01 | 0.38 ± 0.01 | 1.16 ± 0.04 |
|  | Cytosol (13, 3) | 0.44 ± 0.01 | 0.42 ± 0.01 | 1.36 ± 0.09 |
| CBD-GFP | UCV (11, 3) | 0.44 ± 0.01 | 0.43 ± 0.01 | 1.08 ± 0.03 |
|  | Cytosol (12, 3) | 0.44 ± 0.01 | 0.42 ± 0.01 | 1.14 ± 0.03 |
| GFP-GCN4-CBD | UCV (6, 2) | 0.43 ± 0.01 | 0.38 ± 0.01 | 1.28 ± 0.11 |
|  | Cytosol (6, 2) | 0.44 ± 0.01 | 0.40 ± 0.01 | 1.44 ± 0.12 |
| GFP | Cytosol (8, 3) | 0.44 ± 0.01 | 0.40 ± 0.01 | 1.39 ± 0.05 |
